# Monosodium Glutamate Supplementation Improves Bone Status in Mice Under Moderate Protein Restriction

**DOI:** 10.1002/jbm4.10224

**Published:** 2019-09-16

**Authors:** Anne Blais, Gael Y Rochefort, Manon Moreau, Juliane Calvez, Xin Wu, Hideki Matsumoto, François Blachier

**Affiliations:** ^1^ UMR PNCA, AgroParisTech, INRA Université Paris‐Saclay Paris France; ^2^ EA 2496, Dental School Faculty Université Paris Descartes Montrouge France; ^3^ Key Laboratory of Agro‐ecological Process in Subtropical Region, Institute of Subtropical Agriculture Chinese Academy of Sciences Changsha China; ^4^ Institute for Innovation Ajinomoto Co. Inc. Kawasaki Japan

**Keywords:** BONE μCT, BONE TURNOVER, COLLAGEN SYNTHESIS, MONOSODIUM GLUTAMATE, DIETARY PROTEIN RESTRICTION, MICE MODEL

## Abstract

Adequate protein intake during development is critical to ensure optimal bone gain and to attain a higher peak bone mass later. Using a mild protein restriction model in Balb/C mice consuming 6% of their total energy intake as soy protein (LP‐SOY)—for which we observed a significantly lower femoral cortical thickness, bone volume, trabecular number, and thickness reduction—we evaluated the effects of monosodium glutamate (MSG) supplementation at different concentrations (0.5, 1, 5, 10, and 20 g/kg of diet) on bone characteristics in LP‐SOY‐fed mice. After 6 and 12 weeks, LP‐SOY‐fed mice had lower BMD and reduced body weight related to lower lean mass, which was associated with a reduced IGF‐1 level. The negative effect of the LP‐SOY diet on BMD correlated with impaired bone formation. MSG supplementation, at 5, 10, and 20 g/kg of diet, and PTH injection, used as a positive control, were able to improve BMD and to increase osteoblast activity markers (P1NP and osteocalcin), as well as glutamine plasma concentration. An analysis of bone microarchitecture found that cortical bone was less sensitive to protein restriction than trabecular bone, and that MSG ingestion was able to preserve bone quality through an increase of collagen synthesis, although it did not allow normal bone growth. Our study reinforces the view that glutamate can act as a functional amino acid for bone physiology and support clinical investigation of glutamate supplementation in adults characterized by poor bone status, notably as a result of insufficient protein intake. © 2019 The Authors. *JBMR Plus* published by Wiley Periodicals, Inc. on behalf of American Society for Bone and Mineral Research.

## Introduction

Monosodium glutamate (MSG) is a flavor enhancer used worldwide. Added glutamate amounts approximately 0.4 g/day in Europe and 1.5 g/day in Asia.^1^ The average glutamate intake from dietary sources (proteins and free glutamate) is between 10 and 20 g/day.^2^ Glutamate, apart from its role as a quantitatively major amino acid precursor for protein biosynthesis, is a precursor of compounds that play many roles in cell metabolism, regulation of cell metabolism, and cell physiology in mammals including humans.^3^, ^4^ In addition, glutamate is involved in several aspects of taste and gastro‐intestinal physiology.^5^, ^6^ Finally, glutamate is well known to be an excitatory signaling molecule in the central nervous system,^7^ and to act as an energy substrate in many cell types.^8^, ^9^

Regarding the implication of glutamate in bone physiology, recent in vitro studies have suggested a physiological role for this amino acid in the regulation of bone mass. Glutamate, as a so‐called functional amino acid, has been suggested to play a role in bone formation and mass maintenance.^10^ In addition, glutamate has been implicated in osteoblast and osteoclast differentiations.^11^, ^12^ Glutamate displays a mitogenic effect on osteoblasts,^13^ and prevents decreased BMD in femur and tibia after systemic administration.^14^ A recent study also suggests that high glutamate concentration can promote differentiation and activation of osteoblasts.^15^ Moreover glutamate signaling involves both binding to specific receptors and its transport in osteoblasts.^11^, ^16^, ^17^, ^18^ However, from in vivo experiments, it appears that virtually all the enteral glutamate in the diet is metabolized by the gut during absorption; therefore relatively large doses of glutamate in supplements have to be used to increase the glutamate concentration in portal blood, and to a lower extent in arterial blood,^19^, ^20^, ^21^ which puts into question the way glutamate from dietary origin would contribute to intervene in bone physiology and metabolism.

Overall, bone mass and bone quality determine bone strength. BMD and bone microarchitecture depend on osteoblast and osteoclast activity to maintain adequate bone remodeling. For instance, low bone mass can be associated with estrogen deficiency which increases bone resorption over bone formation,^22^ or with inadequate protein intake,^23^ that reduces bone formation. The deleterious consequences of insufficient protein intake are numerous,^16^, ^24^ and both protein quantity and quality have been shown to affect bone quality and muscle mass under moderate protein restriction. We have previously shown that in mice fed a low protein diet (6% of the total energy being supplied by soy protein), had reduced body weight related to a lower lean mass and reduced BMD when compared with mice receiving a sufficient protein supply.^25^

When protein intake is lower than the estimated average requirement, as observed in specific groups of individuals such as the elderly, notably in cases of hospitalization,^26^ and in younger individuals from developing countries,^27^ the utilization of dietary supplements in the form of dietary protein and/or amino acids including glutamate represents a nutritional strategy that may improve osteoblast activity and bone status.

In that overall context, the aim of the present study was to identify the modifications of biological parameters associated with the beneficial effect of glutamate used in its monosodium salt form (MSG). To investigate the effect of different concentrations of MSG on bone, we used a model incorporating mice under mild protein restriction (6% of total energy resulting from soy protein), that was recently implemented in our laboratory. In this experimental model, reduced lean body mass and bone formation were observed,^24^ thus appearing convenient for testing the effect of MSG on osteoblast activity. We have included mice fed a normal protein (NP) control diet (with protein representing 20% of total energy), and seven groups of mice fed a soy protein‐restricted isocaloric diet (low protein [LP]; protein representing 6% of total energy) and receiving different amount of MSG. An additional group of animals used as a positive control received a daily PTH (1‐34 PTH) injection.

## Subjects and Methods

### Animals

Eight‐week‐old Balb/C female mice (Harlan) were housed at 22°C under a 12/12‐hour light cycle. Initially, five animals per cage were used for the first week of habituation. The mice were then moved to individual cages for another week. During the 2 weeks of habituation, the mice were fed a standard AIN‐93M diet containing 20% of total energy as soy protein. The soy protein used in our study did not contain any phytoestrogens to avoid interference with bone metabolism. The design of this study was approved by the French Government (APAFIS#10768‐20170521316215762v2). After the habituation period, 10‐week‐old mice were divided into eight groups of 12 animals.

One group stayed on the 20% soy protein diet used as a NP control group and the seven other groups were shifted to a low protein (LP) diet containing only 6% of total energy as soy protein. One group of mice, which was shifted to the 6% soy protein diet, was also treated for 5 days per week with a s.c. injection of 40 μg/kg of 1‐34 PTH (Sigma‐Aldrich, Lyon, France) as an anabolic control (LP + PTH). Different concentrations of MSG (2%, 1%, 0.5%, 0.1%, and 0.05%) were added to the diet, and the groups were identified, respectively, as: LP +2%, LP +1%, LP +0.5%, LP +0.1%, and LP +0.05% MSG. The last group (control group) was shifted to the 6% soy protein diet (P6 soy) and did not receive MSG, but was supplemented with an iso‐nitrogenous amount of alanine and an iso‐amount of sodium in the form of NaCl (Table 1). To maintain an equal amount of energy intake in the NP and LP diets, protein was replaced by starch and sucrose in the 6% protein diets. To avoid the protein leverage effect in our study and to allow equal energy consumption, we pair‐fed all the LP groups as opposed to the NP control group. Then, the food consumption of the NP soy group was measured every day to determine the amount of diet for the other groups. The LP groups not receiving PTH were injected daily with saline.

**Table 1:** Composition of the Diet

Ingredients (g/kg of diet)	NP	LP	LP + MSG 2%	LP + MSG 1%	LP + MSG 0.5%	PL + MSG 0.1%	LP + MSG 0.05%	LP + PTH
Soy protein^a^	183	51	51	51	51	51	51	51
MSG^b^	0	0	20	10	5	1	0.5	0
Alanine^b^	0	10.47	0	0	0	0	0	0
Nacl^d^	0	6.90	0	3.45	5.18	6.55	6.72	6.90
Corn starch^c^	584	698	698	698	698	698	698	698
Sucrose^e^	95	114	114	114	114	114	114	114
Soybean oil^f^	40	40	40	40	40	40	40	40
Alpha celluose^g^	50	50	50	50	50	50	50	50
AIN 93M mineral mix^h^	35	35	35	35	35	35	35	35
AIN 93M Vitamins^h^	10	10	10	10	10	10	10	10
Choline^i^	2.3	2.3	2.3	2.3	2.3	2.3	2.3	2.3

NP = normal protein; LP = low protein; MSG = monosodium glutamate.

a MP Biomedicals, Irvine, CA, USA.

b Ajinomoto, Tokyo, Japan.

c Cargill, Minnisota, MN, USA.

d Sigma Aldrich, Lyon, France.

e Cristalco, Paris, France.

f Lesieur, Asnières‐sur‐Seine, France.

g Prat Dumas, Couze‐Saint Front, France.

h ICN Pharmaceuticals, Orsay, France.

i Jefo Nutrition, Saint‐Hyacinthe, Quebec, Canada.

### Body composition

The body composition (fat mass and lean mass) was measured at the beginning, after 3 and 6 weeks, and at the end of the study by DEXA, using a Lunar PIXImus densitometer (DEXA‐GE PIXImus; GE Lunar Corp., Madison, WI, USA). The stability of the device was controlled by the measurement of a phantom before each session. The mice were anesthetized by isoflurane inhalation during the measurement. Analysis of the images was performed with the software provided with the device (Lunar PIXImus v2.10; GE Lunar Corp.), using autothresholding. After 12 weeks, the night before anesthesia, the mice were starved overnight; a meal of 1 g was given in the morning 90 min before anesthesia with isoflurane. Blood was drawn by cardiac puncture and the mice were immediately decapitated. Body composition was determined by dissection of the liver, uterus, spleen, kidneys, and pancreas. Four white adipose tissue pads (periovarian, retroperitoneal, mesenteric, and total subcutaneous), interscapular brown adipose tissue, and the carcass (muscle and bone) were removed and weighed.

### Determination of free amino acid profile in plasma

Free amino acids profile in the plasma was measured using ultra‐high‐performance liquid chromatography (UHPLC). Blood was collected 90 min after ingestion of a 1 g meal. Norvaline was added to plasma as an internal standard. Plasma was first deproteinized with sulfosalicylic acid (10 g/L), stored at 4°C and then centrifuged at 3000*g* (4°C) for 30 min. The supernatants were centrifuged for 5 min at 12,000*g* and 10 μL was derivatized using the AccQ Tag Ultra Derivatization Kit (Waters SAS, Guyancourt, France) according to the manufacturer's instructions. UHPLC analyses were performed on an Acquity UPLC H‐Class system with a PDA detector (Waters SAS).

### Isolation and determination of the femur protein fraction

The left femur of each mouse was cleaned of muscles and crushed with a scalpel in an ice cold buffer (50mM Tris‐HCl (pH 7.4), 50mM NaF, 10mM β‐glycerophosphate disodium salt, 1mM EDTA, 1mM EGTA, and 1mM activated Na_3_VO_4_ (all chemicals were obtained from Sigma‐Aldrich), including a complete protease inhibitor cocktail tablet (Roche, West Sussex, UK) used at 10 μL/μg tissue. The homogenates were centrifuged for 10 min, and the pellets were solubilized in 0.3M NaOH for 24 hours. The solubilized protein fraction was separated by centrifugation. A second extraction with NaOH was performed, followed by a subsequent extraction with 0.5M acetic acid. The solubilized fractions were pooled and precipitated with 1M perchloric acid. The pellets were dried and weighted. Pellets (3 mg) were hydrolyzed using HCl 6 N for 24 hours, dried and resuspended in water (100 μL). For the amino acid analysis, the samples were derivatized using the AccQ Tag Ultra Derivatization Kit (Waters SAS) and measured using UHPLC as plasma samples.

### Biochemical analysis

Blood from fasted animals was collected from the tail, and 100 μL of blood were collected after 6 and 12 weeks. N‐terminal propeptides of type I procollagen (PINP) and C‐terminal crosslinking telopeptides of type I collagen (CTx) were measured by enzyme immunoassay (EIA) according to the manufacturer's instructions (Immunodiagnostic Systems, Bolden Business Park, UK). Plasma osteocalcin (OC) and total IGF‐1 levels were determined by ELISA after inactivation of the IGF binding proteins according to the instructions of the manufacturer (Immunodiagnostic Systems).

### Measurement of microarchitectural parameters

Microarchitecture of the femurs was analyzed using a high‐resolution X‐ray μCT device (Quantum FX Caliper; Life Sciences, Perkin Elmer, Waltham, MA, USA), hosted by the PIPA Platform, EA2496; Montrouge, France. The X‐ray source was set at 90 V and 160 μA. Samples up to 65 mm in diameter and 200 mm in length were imaged with a full 3D high‐resolution, and raw data were obtained by rotating both the X‐ray source and the flat panel detector 360 degrees around the sample, with a rotation step of 0.1 degree (scanning time: 3 min). The corresponding 3600 image projections were then automatically reconstructed (RigakuSW software, Caliper, Newton, MA, USA) into a DICOM (Digital Imaging and Communications in Medicine) stack of 512 files using standard back‐projection techniques (reconstruction time: less than a minute). Multiplanar reconstruction tools allowed the display of grey‐level images in an axial orientation. The lowest grey/dark pixels matched with empty spaces; the highest grey/bright pixels represented the densest mineralized tissues. The scans for trabecular bone were initiated from the distal femoral growth plate moving proximally along 50 slices, then 100 slices were analyzed to eliminate the primary spongiosa. The different aspects of trabecular bone were quantified at a 3D isotropic voxel size of 10 × 10 × 10 μm^3^. The structural indices included the ratio of the segmented bone volume to the total volume of the region of interest (BV/TV, %), trabecular number (Tb.n, 1/mm), and trabecular separation (Tb.Sp, μm), which were calculated by the Mean Intercept Length method, structure model index (SMI) and degree of anisotropy (DA). Trabecular thickness (Tb.Th, μm) was calculated on 3D images using CtAn Skyscan software (Bruker, Kontich, Belgium) through a method described by Hildebrand and Ruegsegger.^28^, ^29^ Cortical thickness and diameters (mm) were measured directly and manually on the image at the middiaphysis level. Cortical porosity (Ct.Po, %), cortical bone area (B.Ar, mm^2^), and moment of inertia (mm^4^) were measured at the middiaphysis level as previously described.^30^ All details of these measurements have been published by Lespessailles and colleagues.^31^

### Bone characteristics

The left femur of each mouse was cleaned of muscles and dried overnight at 110°C, weighed, then ashed at 550°C for 48 hours, and the weight of the ash was then evaluated. The difference between the dry weight and the ash gives an indication of the protein fraction in bone.

### Statistical analysis

Results were expressed as means ± SD together with the number of independent experiments. Results were compared using a one‐way ANOVA and a Tukey multiple comparison test to assess the effect of treatment. Significance was established at *P* < 0.05. All statistical analyses were performed using Prism Version 6.05 (GraphPad Software Inc., La Jolla, CA, USA).

## Results

### Effects of MSG on body composition

As indicated in Table 2, the body weight of mice ingesting the LP diet for 6 and 12 weeks remained almost unchanged when compared with the initial weight, whatever was the amount of MSG used as dietary supplement.

**Table 2:** Effect of Restricted Protein Diet With Increasing Amounts of Monosodium Glutamate (MSG) on Body Weight and Body Composition

	NP	LP	LP + 0.05%	LP + 0.1%	LP + 0.5%	LP + 1%	LP + 2%	LP + PTH
Initial weight (g)	20.1 ± 0.8	19.9 ± 0.9	20.0 ± 0.7	20.0 ± 1.1	19.6 ± 1.0	20.4 ± 1.1	19.8 ± 1.1	20.1 ± 0.8
Weight gain after 6W (g)	1.7 ± 0.2^a^	− 0.2 ± 0.5^b^	− 0.1 ± 0.4 ^b^	− 0.2 ± 0.5 ^b^	− 0.4 ± 0.5 ^b^	− 0.1 ± 0.5 ^b^	− 0.2 ± 0.6 ^b^	− 0.2 ± 0.6 ^b^
Weight gain after 12W (g)	3.8 ± 0.4 ^a^	− 0.2 ± 0.4 ^b^	0.4 ± 0.5 ^b^	0.8 ± 0.6 ^b^	0.7 ± 0.6 ^b^	0.0 ± 0.5 ^b^	0.1 ± 0.8 ^b^	0.1 ± 0.3 ^b^
Lean body mass gain after 6W (g)	0.9 ± 0.3 ^a^	− 0.7 ± 0.2 ^b^	− 0.6 ± 0.3 ^b^	− 0.7 ± 0.2 ^b^	− 0.3 ± 0.2 ^b^	− 0.3 ± 0.2 ^b^	− 0.4 ± 0.4 ^b^	− 0.2 ± 0.3 ^b^
Lean body mass gain after 12W (g)	1.8 ± 0.2 ^a^	− 0.2 ± 0.4 ^b^	− 0.3 ± 0.2 ^b^	− 0.3 ± 0.2 ^b^	− 0.2 ± 0.4 ^b^	− 0.2 ± 0.3 ^b^	− 0.4 ± 0.5 ^b^	0.0 ± 0.3 ^b^
Fat mass gain after 6W (g)	0.7 ± 0.1	1.0 ± 0.2	1.5 ± 0.3	1.2 ± 0.3	1.2 ± 0.2	1.4 ± 0.3	1.4 ± 0.4	1.0 ± 0.2
Fat mass gain after 12W (g)	2.0 ± 0.1	2.6 ± 0.4	2.1 ± 0.3	2.8 ± 0.4	2.6 ± 0.3	2.0 ± 0.1	1.9 ± 0.2	2.5 ± 0.2

The mice were fed for 6 and 12 weeks with either normal protein (NP) diet (20% soy protein) or with a 6% soy protein diet (low protein [LP]) without or with increasing amounts of MSG, and for the positive anabolic control, with 1‐34PTH injection (LP + PTH). Gain of lean and fat mass was evaluated by DEXA between the beginnings and after 6 weeks (6W) or 12 weeks (12W). Data are expressed as mean ± SD, *n* = 12. Means that are significantly different (*p* < 0.05) have different letters.

**Figure 1:**
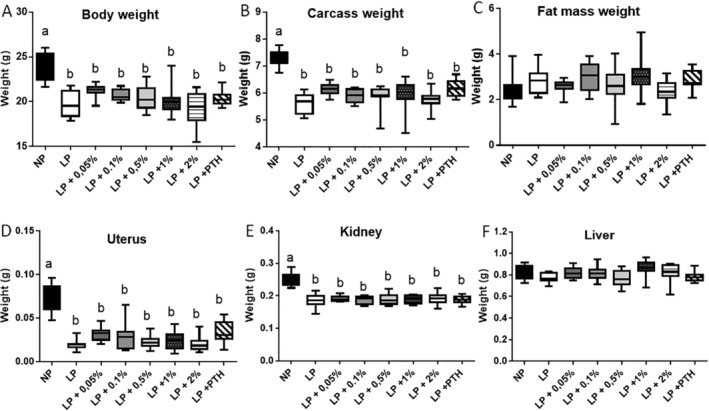
Effect of restricted protein diet with increasing amounts of monosodium glutamate (MSG) on body weight and body composition. The mice were fed for 12 weeks with either normal protein (NP) diet (20% soy protein) or with a 6% soy protein diet (low protein [LP]), without or with increasing amounts of MSG, and for the positive anabolic control with 1‐34 PTH injection (LP + PTH). Body and organs were weighed immediately after sacrifice. (*A*) Body weight. (*B*) Carcass weight. (*C*) Total fat mass. (*D*) Uterus weight. (*E*) Kidney weight. (*F*) Liver weight. Data are presented as box and whiskers, *n* = 12 per group. Each group is compared with the others by a one‐way ANOVA on repeated measures followed by a Tukey post hoc test. Means that are significantly different (*p* < 0.05) according to the Tukey multiple comparison test have different letters.

According to DEXA measurements, the LP diet appeared to affect only the lean mass, as the fat mass gain was similar to the values recorded in the NP group. When the animals receiving the LP diet were treated with PTH, no effect of this anabolic hormone on body weight and composition was measured. Final body and organ weights are presented in Fig. 1. The final body weight of the mice in all the LP groups was significantly lower than the weight measured in the control NP group (Fig. 1 *A*). The significant lower body weight of LP animals was correlated with a lower carcass weight (Fig. 1 *B*), but not with a decrease of the total fat mass, this parameter was similar for the NP and LP groups (Fig. 1 *C*). This confirms that a moderate reduction of protein intake has a significant impact only on the lean body mass gain. Analysis of the weight of different organs showed that the LP diet also reduced uterus (Fig. 1 *D*) and kidney (Fig. 1 *E*) weight, but did not modify liver weight (Fig. 1 *F*). The MSG supplementation or PTH injection did not have any significant effect on those parameters. As the LP diet reduces the lean body mass, muscular strength was evaluated by using the grip test. Values of 85.7 ± 2.7 versus 83.3 ± 1.9 Newton were measured for the NP and LP group, respectively, thus indicating that muscular strength appears proportional to the body weight of the animals.

### Effect of MSG on free amino acids in the plasma

We evaluated in mice the plasma amino acid concentrations 90 min after ingestion of a 1‐g meal without or with different doses of MSG. Table 3 shows that, when compared with values recorded in the NP group, the ingestion of the LP diet decreased plasma concentration of all amino acids measured except, as expected, alanine. This latter result is because the LP control group was supplemented with an iso‐nitrogenous amount of alanine. Ingestion of the diet containing 1% and 2% of MSG increased significantly the plasma concentration of glutamine, by 25% and 49%, respectively, when compared with the concentrations measured in the control LP diet group. The highest concentration of MSG used was also able to increase the alanine plasma concentration by 30% compared with the concentrations measured in the control LP diet group. All the other amino acid concentrations in plasma, notably the glutamate concentration, remained unchanged whatever the dose of MSG used. The treatment of protein‐restricted mice with the lowest MSG concentration or PTH had no significant effect on any of the circulating amino acid concentrations measured in plasma (data not shown).

**Table 3:** Plasma Amino Acid Concentration After Ingestion of a 1‐g Meal

	LP	LP + 0.5%	LP + 1%	LP + 2%	NP
Ala	553.3 ± 33.7^a^	555.9 ± 57.1^a^	526.2 ± 48.7^a^	718.4 ± 97.8^b^	575.4 ± 71.4^a^
Arg	50.9 ± 7.7 ^a^	51.9 ± 4.7 ^a^	54.1 ± 4.0 ^a^	48.3 ±3.4 ^a^	125.4 ±9.9 ^b^
Asn	60.1 ± 9.8 ^a^	60.0 ± 4.2 ^a^	59.1 ± 4.3 ^a^	71.3 ± 9.4 ^a^	149.07 ± 19.7 ^b^
Asp	2.69 ± 0.78 ^a^	3.01 ± 0.72 ^a^	3.12 ± 1.02	3.24 ± 1.27 ^a^	3.98 ± 1.45 ^b^
Gln	343.3 ± 38.8^a^	357.7 ± 26.5^a^	428.9 ± 26.4^a,b^	512.1 ± 68.2^b^	684.8 ± 91.6^c^
Glu	57.8 ± 7.0 ^a^	58.2 ± 3.2 ^a^	59.9 ± 4.8 ^a^	54.8 ± 3.2 ^a^	99.9 ± 13.7 ^b^
Gly	172.5 ± 20.8 ^a^	171.2 ± 11.0 ^a^	195.6 ± 13.4 ^a^	184.3 ± 13.3 ^a^	259.3 ± 24.8 ^b^
His	51.0 ± 9.32 ^a^	51.1 ± 3.38 ^a^	54.4 ± 2.8 ^a^	55.5 ± 5.0 ^a^	75.54 ± 6.4 ^b^
Ile	35.6 ± 4.2 ^a^	34.0 ± 3.8 ^a^	35.8 ± 2.8 ^a^	38.7 ± 4.8 ^a^	119.6 ± 11.6 ^b^
Leu	37.1 ± 6.4 ^a^	37.9 ± 4.8 ^a^	36.9 ± 4.6 ^a^	41.1 ± 5.1 ^a^	160.1 ± 15.1 ^b^
Lys	211.0 ± 23.6 ^a^	201.6 ± 15.1 ^a^	204.9 ± 26.8 ^a^	198.0 ± 15.2 ^a^	369.09 ± 38.8 ^b^
Met	26.2 ± 4.0 ^a^	26.6 ± 2.4 ^a^	28.2 ± 2.8 ^a^	29.2 ± 2.0 ^a^	65.1 ± 5.3 ^b^
Phe	41.8 ± 6.2 ^a^	40.7 ± 4.5 ^a^	42.4 ± 4.1 ^a^	44.7 ± 3.3 ^a^	87.5 ± 6.8 ^b^
Pro	92.7 ± 8.5 ^a^	93.3 ± 6.8 ^a^	92.2 ± 8.2 ^a^	105.2 ± 11.7 ^a^	165.8 ± 16.2 ^b^
Ser	139.8 ± 11. 6 ^a^	133.7 ± 8.8 ^a^	137.5 ± 11.8 ^a^	127.0 ± 16.0 ^a^	214.8 ± 22.0 ^b^
Thr	113.8 ± 15.2 ^a^	115.4 ± 9.2 ^a^	127.8 ± 7.0 ^a^	127.9 ± 10.2 ^a^	329.9 ± 27.8 ^b^
Trp	57.7 ± 10.6 ^a^	58.1 ± 8.0 ^a^	63.1 ± 12.6 ^a^	57.5 ± 8.1 ^a^	121.8 ± 13.8 ^b^
Tyr	33.9 ± 4.9 ^a^	26.1 ± 3.8 ^a^	29.5 ± 5.4 ^a^	29.9 ± 5.8 ^a^	97.6 ± 9.2 ^b^
Val	76.8 ± 4.9 ^a^	80.8 ± 9.0 ^a^	86.6 ± 4.9 ^a^	87.6 ± 6.5 ^a^	304.1 ± 31.1^b^

The mice were fed with either the normal protein (NP) diet (20% soy protein) or the low protein diet (LP, 6% soy protein) without or with increasing amounts of MSG, and received a 1‐g‐test meal. Blood was recovered 90 min after the ingestion of the meal for amino acid analysis. In the LP group (not receiving MSG), mice were supplemented with an iso‐nitrogenous amount of alanine. Data are expressed as mean ± SD, *n* = 12. Means that are significantly different (*p* < 0.05) have different letters.

### Effects of MSG on bone mineral density

**Figure 2:**
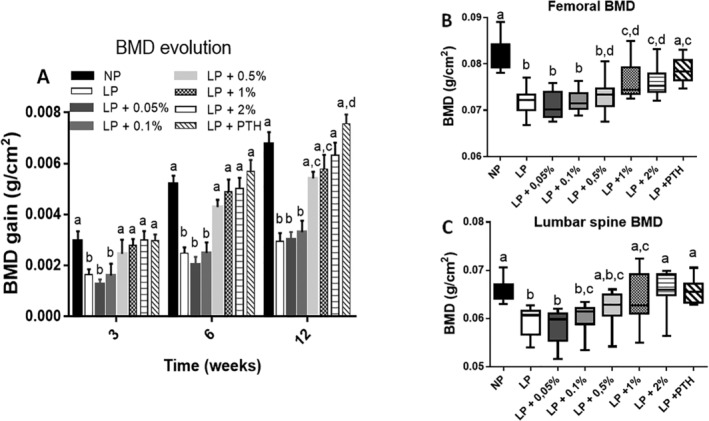
Effect of restricted protein diet with increasing amounts of monosodium glutamate (MSG) on whole body, femoral and lumbar spine BMD. The mice were fed for 12 weeks with either normal protein (NP) diet (20% soy protein) or with a 6% soy protein diet (low protein [LP]) without or with increasing amounts of MSG, and for the positive anabolic control with 1‐34 PTH injection (LP + PTH). (*A*) Evolution of whole‐body BMD as function of time. BMD gain was compared with the T0 values. Values are expressed as mean ± SD, *n* = 12. (*B*) Effect after 12 weeks of treatment with increasing amounts of MSG or PTH on femoral BMD. (*C*) Effects after 12‐week treatment with increasing amounts of MSG or PTH on lumbar spine BMD. Data are presented as box and whiskers for femoral and lumbar spine BMD, with *n* = 12 per group. Each group is compared with the others by a one‐way ANOVA on repeated measures followed by a Tukey post hoc test. Means that are significantly different (*p* < 0.05) according to the Tukey multiple comparison test have different letters.

BMD gain, as a function of time, is shown on Fig. 2 *A*. The LP diet reduced significantly BMD gain after 3, 6, and 12 weeks when compared with the BMD measured in NP animals. Although the two lowest concentrations of MSG used for supplementation were not able to improve BMD gain, the 0.5%, 1.0%, and 2.0 % doses were able to significantly improve this parameter as soon as 3 weeks after treatment. As previously shown by Rouy and colleagues^25^ s.c. injection of PTH was able to preserve the BMD in our model, and the MSG used at 1% and 2% doses was nearly as efficient as PTH to preserve the BMD. Analysis of femoral and lumbar spine BMD (Fig. 2 *B*,*C*), after supplementation with MSG for 3 months shows that MSG ingestion, when used at 1% and 2% doses, was more efficient to preserve BMD at the vertebral than at the femoral level. Indeed, the 2% dose of MSG was as efficient as PTH injection to maintain lumbar spine BMD.

### Effects of MSG on IGF‐1 and bone‐remodeling marker plasma concentration

**Figure 3:**
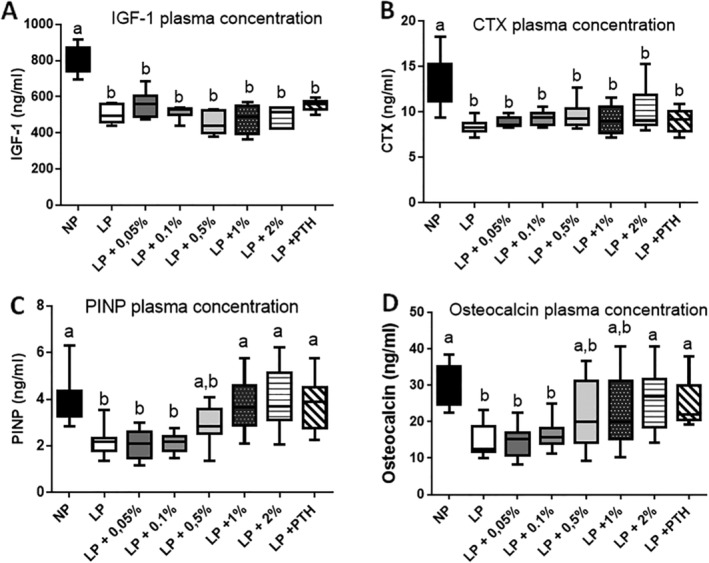
Effect of restricted protein diet with increasing amounts of monosodium glutamate (MSG) on IGF‐1, CTX, PINP, and osteocalcin plasma concentrations. The mice were fed for 12 weeks with either normal protein (NP) diet (20% soy protein) or with a 6% soy protein diet (low protein [LP]) without or with increasing amounts of MSG, and for the positive anabolic control, with 1‐34 PTH injection (LP + PTH). Then IGF‐1 (*A*) and the bone remodeling markers CTX (*B*), PINP (*C*), and osteocalcin (*D*) were measured in the plasma. Data are presented as box and whiskers, *n* = 12 per group. Each group is compared with the others by a one‐way ANOVA on repeated measures followed by a Tukey post hoc test. Means that are significantly different (*p* < 0.05) according to the Tukey multiple comparison test have different letters.

As indicated in Fig. 3 *A*, the LP diet reduced the IGF‐1 plasma concentration by nearly 50%. MSG supplementation or PTH injection did not have any effect on the IGF‐1 level. Plasma concentration of CTX (Fig. 3B), the osteoclast activity marker, was severely decreased when mice ingested the LP diet. MSG supplementation or PTH injection did not have any effect on the CTX level. The LP diet also reduced the plasma concentrations of PINP (Fig. 3C) and osteocalcin (Fig. 3D), two markers of bone formation. However, the positive control PTH and the highest concentration of MSG (2%) were able to increase both markers, suggesting that osteoblast activity was largely restored by MSG supplementation.

### Effects of MSG on bone microarchitecture

**Figure 4:**
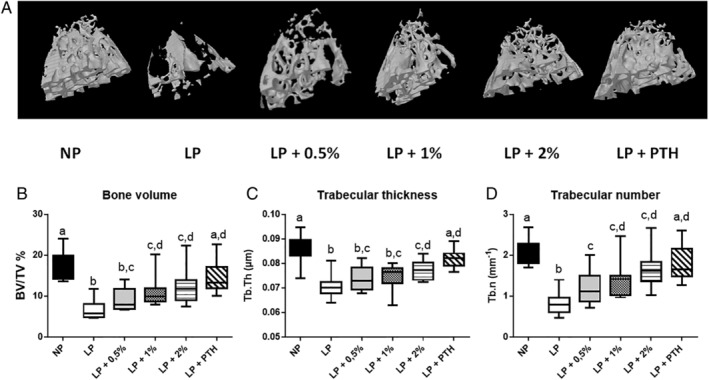
Effect of restricted protein diet with increasing amounts of monosodium glutamate (MSG) on trabecular bone. The mice were fed for 12 weeks with either normal protein (NP) diet (20% soy protein) or with a 6% soy protein diet (low protein [LP]) without or with increasing amounts of MSG, and for the positive anabolic control, with 1‐34 PTH injection (LP + PTH). Then the bone microarchitecture was determined. Typical and representative examples of ex vivo μCT reconstruction of trabecular bone in different conditions (upper panel) and in lower panel comparison of (B) the bone volume to the total volume of the region of interest (BV/TV), (C) trabecular thickness (Tb.Th), and (D) trabecular number (Tb.N) in different conditions.

**Figure 5:**
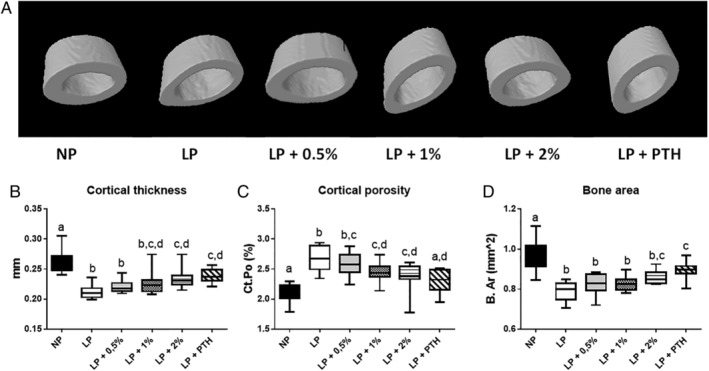
Effect of restricted protein diet with increasing amounts of monosodium glutamate (MSG) on cortical bone. The mice were fed for 12 weeks with either normal protein (NP) diet (20% soy protein) or with a 6% soy protein diet (low protein [LP]) without or with increasing amounts of MSG, and for the positive anabolic control, with 1‐34 PTH injection (LP + PTH). Then the bone microarchitecture was determined. Typical and representative examples of ex vivo μCT reconstruction of cortical bone in different conditions (upper panel) and in lower panel comparison of (B) cortical thickness, (C) cortical porosity (Ct.Po), and (D) bone area (B.Ar) in different conditions.

As indicated in Figs. 4 and 5, and Table 4, the LP diet reduced both trabecular (Fig. 4A) and cortical bone (Fig. 5A) microarchitecture. However, trabecular bone was found to be more sensitive than cortical bone to a LP diet. DEXA and μCT data were in agreement with this latter result, identifying a negative effect of the LP diet on bone microarchitecture. MSG supplementation from 0.5% to 2% dose‐dependently improved bone as shown on μCT and, as previously shown, PTH injection, used as positive control, was able to preserve bone as seen on μCT. Lower doses of MSG were insufficient to preserve bone microarchitecture (data not shown).

**Table 4:** Effect of Restricted Protein Diet With Increasing Amounts of Monosodium Glutamate (MSG) on Bone Microarchitecture Characteristics and Hydroxyproline Content

Trabecular bone parameters						
	NP	LP	LP + 0.5%	LP + 1%	LP + 2%	LP + PTH
Cortical bone parameters						
BS/BV (mm^–1^)	44.66 ± 1.17^a^	59.72 ± 1.21^b^	55.72 ± 1.68^b,c^	51.55 ± 1.68^c^	49.81 ± 1.05^a^	47.35 ± 0.78^a^
Tb.Sp (μm)	274 ± 6^a^	409 ± 14^b^	374 ± 15^b,c^	336 ± 13^a,c^	312 ± 10^a^	294 ± 9^a^
SMI	2.12 ± 0.04^a^	2.64 ± 0.05^b^	2.52 ± 0.07^a,b^	2.50 ± 0.07^a,b^	2.42± 0.08^a,b^	2.28 ± 0.06^a^
DA	1.76 ± 0.06	1.92 ± 0.20	1.83 ± 0.14	1.74 ± 0.08	1.69 ± 0.12	1.73 ± 0.06
Cortical bone parameters						
B.Ar (mm^2^)	0.971 ± 0.020^a^	0.806 ± 0.009^b^	0.840 ± 0.010^b,c^	0.858 ± 0.014^c^	0.856 ± 0.009^c^	0.894 ± 0.011^c^
Av. mom inertia (mm^4^)	0.109 ± 0.003^a^	0.081 ± 0.002^b^	0.091 ± 0.003^b,c^	0.090 ± 0.003^b,c^	0.097 ±0.003^c^	0.099 ± 0.003^c^
Dia ext AP (mm)	1.67 ± 0.03	1.58 ± 0.14	1.61 ± 0.07	1.60 ± 0.06	1.63 ± 0.06	1.61 ± 0.05
Dia ext ML (mm)	1.15 ± 0.04	1.09 ± 0.03	1.12 ± 0.03	1.11 ± 0.03	1.13 ± 0.04	1.10 ± 0.05
Bone characteristics						
Proline mM/mg protein extract	137.2 ± 16.5	125.6 ± 22.2	124.8 ± 30.2	122.2 ± 23.8	137.7 ± 23.4	130.9 ± 14.5
Hydroxyproline mM/mg protein extract	2.8 ± 0.4^a^	0.7 ± 0.1^b^	1.5 ± 0.4^b,c^	2.0 ± 0.5^a,b^	1.9 ± 0.4^a,b^	2.2 ± 0.3^a,c^

The mice were fed for 12 weeks with either a normal protein (NP) diet (20% soy protein) or with a 6% soy protein diet (low protein [LP]) without or with increasing amounts of MSG, and for the positive anabolic control, with 1‐34 PTH injection (LP + PTH), then bone μCT characteristics and hydroxyproline content were determined. Values are expressed as mean ± SD, *n* = 12. Each group was compared with the others by a one‐way ANOVA on repeated measures followed by a Tukey post hoc test. Means that are significantly different (*p* < 0.05) according to the Tukey multiple comparison test have different letters.

BS/BV = bone volume; Tb.Sp = trabecular separation; SMI = structure model index; DA = degree of anisotropy; B.Ar = bone area; Av. mom inertia = average moment of inertia; Dia ext AP = diameter external anteroposterior; Dia ext ML = diameter external mediolateral.

In the femur, the LP diet decreased BV/TV (Fig. 4B), Tb.Th (Fig. 4C), and Th.N (Fig. 4D), and increased BS/BV, Tb.Sp, and SMI, but did not display any effect on DA (Table 5). Ingestion of the LP diet for 12 weeks decreased BV/TV and Tb.N by 63% and 61%, respectively. MSG ingestion dose‐dependently improved trabecular bone characteristics. The highest concentration of MSG used (2%) was almost as efficient as PTH in preserving trabecular bone.

Analysis of the bone microarchitecture characteristics confirmed that a LP diet did have less impact on cortical than on trabecular bone. We report a reduction of 19% of the cortical thickness (Fig. 5B) and an increase in the Ct.Po (Fig. 5C). Moreover, PTH, used as a positive control, or MSG was not able to completely reverse the impact of a LP diet on cortical bone. Indeed, 10% of cortical bone was lost when mice ingested the LP + MSG 2% or LP + PTH diets. We reported the same results for the B.Ar (Fig. 5D) and for the average moment of inertia (Table 5). However, the Ct.Po was restored in the LP + PTH group and was significantly improved in the LP + 2% MSG group.

**Figure 6:**
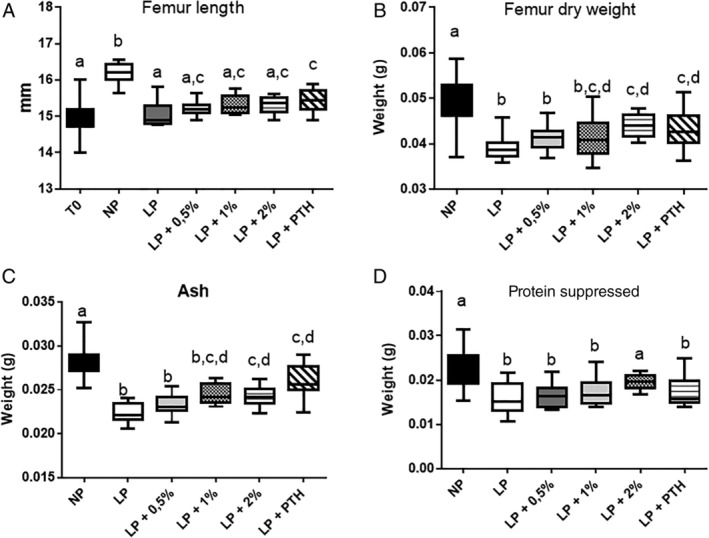
Effect of restricted protein diet with increasing amounts of monosodium glutamate (MSG) on bone characteristics. The mice were fed for 12 weeks with either normal (NP) protein diet (20% soy protein) or with a 6% soy protein diet (low protein [LP]) without or with increasing amounts of MSG, and for the positive anabolic control with 1‐34 PTH injection (LP + PTH). (*A*) Femur length. (*B*) Femur dry weight. (*C)* Ash weight. (*D*) Protein weight. Data are presented as box and whiskers for femoral and lumbar spine BMD, with *n* = 12 per group. Each group is compared with the others by a one‐way ANOVA on repeated measures followed by a Tukey post hoc test. Means that are significantly different (*p* < 0.05) according to the Tukey multiple comparison test have different letters.

To better understand the effect of MSG and PTH, different bone characteristics were analyzed. Femur length measured at the beginning (15.00 mm ± 0.07 mm) and at the end of the experiment, are presented in Fig. 6A. Ingestion of the LP diet arrested bone‐length growth and as shown for the analysis of the cortical bone, the bone length in the LP + 2% and LP + PTH groups increased when compared with the LP group. However, these treatments did not allow for a catch‐up to normal bone length when compared with the NP group. Bone dry weight measurement (Fig. 6B) showed that the LP diet reduced not only bone length, but also bone size, indicating that such a dietary restriction is related not only to a reduction of the mineral part, but also to a reduction of the protein fraction. MSG and PTH were able to improve bone mineral content (Fig. 6C). However, the mineral part of the bone never caught‐up with the NP group. As the calcium content of all the diets was similar, the results emphasize the importance of protein content to maintain bone quality. Evaluation of the protein content (Fig. 6D) by subtraction of the ash component from the dry weight showed that only the highest concentration of MSG was able to increase significantly the bone protein content when the LP diet was ingested.

Finally, bone protein was extracted and the hydroxyproline contents, used as an amino acid specifically found in collagen, were determined. The LP diet lowered the hydroxyproline content, but MSG ingestion and PTH injection were able to increase this content. Regarding proline, which is not specifically found in collagen, we found no modification of this amino acid in bone protein after MSG supplementation.

## Discussion

The data obtained in the present study indicate that MSG, used as a dietary supplement, can largely restore, in a dose‐dependent manner, the impaired bone status in mice receiving a restricted amount of protein in their diet. The effects of MSG on BMD, bone‐remodeling biochemical markers, and microarchitecture characteristics were associated with a dose‐dependent effect on the supplementation of the glutamine plasma concentration that was markedly increased, although glutamate‐circulating concentration remained unchanged. Because of the intense catabolism of glutamate in the intestine,^3^ very large doses of glutamate in supplements are necessary to increase the concentration of this amino acid in the portal vein, and more weakly in arterial blood.^21^ The concentrations of all the other amino acids were not changed after MSG supplementation, with the notable exception of alanine, which was increased by the highest dose of MSG. Such an increase corresponds to the high capacity of intestinal epithelial cells to transaminate glutamate in the presence of pyruvate, allowing alanine and alpha‐ketoglutarate production.^3^ It is then tempting to propose that the effects of MSG on bone would be largely related to its capacity to increase glutamine concentration in blood. There are several arguments in favor of this interpretation. First, glutamate supplementation has been shown to increase glutamine‐circulating concentrations in several experimental models.^32^, ^33^ Second, glutamine represents a major oxidative fuel in osteoblasts, fulfilling an important part of the energy requirement and promoting protein synthesis.^34^ Although out of the scope of the present study, the way by which glutamate supplement increases glutamine plasma concentration is worth a brief discussion. One plausible possibility is that dietary MSG supplementation would increase glutamate intake in enterocytes and thus its intracellular concentration. Because the intestine is well known to represent a major site for glutamine catabolism in the body through glutaminase activity,^35^ and glutaminase in intestinal epithelial cells has been shown to be strongly inhibited by glutamate,^36^ there is a sparing effect of glutamate on glutamine catabolism in the intestines.^21^ Another way to increase glutamine concentration is through the activity of glutamine synthetase. This activity is expressed at very low levels in the intestines,^37^ in contrast with its activity in perivenous hepatocytes.^38^ In other words, glutamate would limit glutamine utilization in the intestines and be released in the portal vein for glutamine synthesis in the liver. Further work is obviously required to decipher the ways by which glutamate increases glutamine plasma concentrations.

The addition of 2% of MSG to the diet was nearly as efficient as the treatment of animals with PTH, thus indicating a robust effect of this amino acid on bone. Interestingly, although both the lumbar spine and femoral BND were improved by MSG supplementation, the effect was more marked at the vertebral than at the femoral level. Given that different skeletal sites do express different sensitivities to estrogen withdrawal or to pharmaceutical treatments, this result was not unexpected.

As previously reported, the LP diet used in our study induced a marked reduction of BMD gain^25^; this decrease was correlated with decreased plasma levels of IGF‐1, CTX, PINP, and OC. IGF‐1 is a growth factor with both endocrine and paracrine/autocrine anabolic actions on bone.^22^ Moreover, its plasma concentration is well‐correlated not only with protein levels, but protein quality as well,^39^ whereas CTX is a marker for bone resorption and PINP and OC are markers for bone formation. Furthermore, the LP diet markedly altered bone microarchitecture; thus confirming that a LP diet for growing mice has severe effects on bone. Our results show that the addition of 2% MSG to the diet preserved bone quality as efficiently as PTH used as a positive control. In a previous study, we have shown that the changes, as seen on μCT, in the femur of mice receiving a LP diet were associated with an increased adipocyte volume in the marrow and to a lower osteoid surface compared with NP and LP + PTH. These results indicate that a PTH injection is able to preserve bone formation. In the present study, we evaluated the osteoblasts activity through the hydroxyproline content in the femur, which is specific for the measurement of bone collagen. Our results indicate that both PTH and MSG were able to preserve osteoblast activity.

The LP diet reduced not only BMD gain, but lean mass gain, as well as uterus and kidney weight, supporting a reduction of protein synthesis not only in bone, but also in other tissue sensitive to protein restriction. However, MSG supplementation, although it improves bone physiology, had no effect on the weight of the lean mass, uterus and kidney. Regarding the inability of MSG and PTH to counteract the negative effect of a LP diet on lean mass, this result is associated with the inability of both treatments to restore a normal IGF‐1 plasma concentration.

Our data show that MSG efficiently preserves BMD, PINP, and OC plasma levels and bone hydroxyproline content; this latter parameter directly correlates with collagen synthesis in bone. This suggests that glutamine specifically stimulates the activity of osteoblasts in bone. These results agree with the μCT analysis: Both MSG and PTH were able to preserve trabecular and cortical bone microarchitecture. However, a detailed analysis of bone characteristics showed that even if MSG and PTH improved bone quality, they did not allow a complete catch‐up of femur growth. Our results are in accordance with a study showing that the bone anabolic effects of PTH were attenuated in rats fed a LP diet.^40^

Although our in vivo data clearly show that MSG supplementation is an efficient way to preserve bone quality in a model where bone quality is greatly compromised, questions remain about the way glutamate exerts its beneficial effect on bone. The fact that MSG failed to increase the IGF‐1 level in the LP animals implies that this amino acid acts on bone independently of this growth factor. There is an intriguing question about the ways glutamate, likely through its capacity to serve as a substrate for glutamine synthesis and as a regulator of glutamine degradation, can preserve bone status without furnishing all the other amino acids required for increased protein synthesis in bone. To answer this important question, it is worth considering that PTH, in the absence of any amino acid given as a supplement, can markedly restore bone quality. In other words, it is likely that glutamine (just like PTH), by stimulating osteoblast activity, increases the uptake of the circulating amino acids needed for increasing the protein synthesis in these cells. However, under moderate protein restriction, even with MSG supplementation, we found that bone length was reduced; hence, MSG favors bone quality at the expense of growth in bone length. As MSG improved the plasma concentration of glutamine but PTH did not, the mechanisms presumably involved for the stimulation of osteoblast activity by MSG must implicate different pathways than the ones involved in the PTH action. Recent studies have shown that stimulating glycolysis in preosteoblasts increases bone formation in vivo.^41^ Our results, which show a good correlation between the preservation of bone quality and the glutamine plasma level, are in agreement with previous in vitro studies showing that glutamine import was required by calvarias osteoblasts for matrix mineralization.^42^ Moreover, previous data have shown that increased glutamine level favors energy metabolism in osteoblasts,^43^ and that decreased glutamine consumption by bone marrow stromal cells in elderly mice has been linked to impaired osteoblast differentiation.^44^ Therefore, glutamine can be an important osteoblast regulator.

## Conclusion

We found that relatively large doses of MSG in cases of moderate protein restriction, likely by allowing an increase in glutamine plasma concentration, stimulate osteoblast activity and improve the altered BMD and bone microarchitecture. Our study thus reinforces the view that glutamate supplementation can be useful in cases of poor bone status. However, more clinical studies are needed on glutamate supplementation in adults with poor bone status as a result of insufficient protein intake.

Disclosures

All authors state that they have no conflicts of interest.
